# Use of Dietary Fibers in Reducing the Risk of Several Cancer Types: An Umbrella Review

**DOI:** 10.3390/nu15112545

**Published:** 2023-05-30

**Authors:** Jun Hu, Junjing Wang, Yuxing Li, Kun Xue, Juntao Kan

**Affiliations:** 1 Department of Nutrition and Food Hygiene, School of Public Health, Fudan University, Shanghai 200032, China; 22211020134@m.fudan.edu.cn; 2 Nutrilite Health Institute, Shanghai 201203, China; junjing.wang@amway.com; 3 Department of Preventive Medicine and Health Education, School of Public Health, Fudan University, Shanghai 200032, China; 19301020087@fudan.edu.cn

**Keywords:** dietary fiber, cancer, protective effects, meta-analysis, umbrella review

## Abstract

(1) Background: Numerous meta-analyses have shown that a high intake of dietary fiber plays a protective role in preventing the development of various types of cancer. However, previous studies have been limited by focusing on a single type of dietary fiber and variations in outcome measures, which may not be effectively applied to provide dietary guidance for the general population. (2) Object: We summarized the meta-analysis of dietary fiber and cancer, and provided references for residents to prevent cancer. (3) Methods: Systematic search of relevant meta-analyses on the association between dietary fiber and cancer occurrence in PubMed, Web of Science and other databases was conducted from the time of database construction to February 2023. The method logical and evidence quality assessments were performed by applying the criteria in the “A Measurement Tool to Assess Systematic Reviews-2” (AMSTAR2) scale and the World Cancer Research Fund/American Institute for Cancer Research (WCRF/AICR) Expert Report, respectively. (4) Results: Our analysis included 11 meta-analyses, and the AMSTAR 2 assessment revealed that the overall methodological quality was suboptimal, with two key items lacking sufficient information. Nonetheless, our findings indicate that a high intake of dietary fiber is associated with a reduced risk of several types of cancer, including esophageal, gastric, colon, rectal, colorectal adenoma, breast, endometrial, ovarian, renal cell, prostate, and pancreatic cancers. The majority of these associations were supported by a “probable” level of evidence. (5) Conclusions: Dietary fiber intake has different protective effects on different cancers.

## 1. Introduction

According to a 2019 report from the World Health Organization (WHO), cancer was the leading or second-leading cause of death in 112 countries and the third- or fourth-leading cause of death in 23 countries [1]. The impact of cancer is not limited to causing suffering for families but also obstructs socioeconomic development. In the latest report released by the IARC in 2020, breast cancer topped the list of incidences, while colorectal, prostate, gastric, and esophageal cancers were also ranked among the top 10 with high mortality rates [2]. Therefore, confronting the economic burden of treating cancer and the low cure rate, effective prevention is considered the most efficient and cost-effective strategy for cancer control.

Epidemiological research has consistently demonstrated the significant role of dietary factors in preventing cancer, with dietary fiber being of particular interest [3]. The European Food Safety Authority (EFSA) defined dietary fiber as indigestible carbohydrates and lignin. EFSA provided an extensive list of substances that make up dietary fiber, including non-starch polysaccharides, cellulose, pectin, hydrocolloids, oligofructose, and “resistant starch” [4]. Based on their water solubility, dietary fiber can be classified as soluble or insoluble. Soluble dietary fiber is primarily found in vegetables and fruits, while insoluble dietary fiber is primarily found in cereals and whole grain products. Despite being present in many common foods, dietary fiber intake often falls short of the recommended daily intake. According to a survey by Chinese scholars, the average dietary fiber intake among Chinese adults aged 20 years and above was 9.7 g/day in 2015, with a decreasing trend [5]. Similarly, adults in European countries consume 18–24 g/day of dietary fiber for men and 16–20 g/day for women, which is below the recommended daily intake [6]. While some studies have shown that increased dietary fiber intake can protect against certain types of cancer, such as rectal and breast cancer [7,8], other epidemiological studies have produced conflicting or weak results [9,10,11]. Given the contradictory findings regarding the protective effects of dietary fiber on cancer, this study aims to provide reliable, evidence-based medicine for clinical practice by conducting a systematic review of relevant meta-analyses examining the association between dietary fiber and cancer.

## 2. Methods

### 2.1. Literature Search

We conducted a comprehensive literature search from the establishment of the database to February 2023 by screening PubMed, Web of Science, Embase, The Cochrane Library, China Knowledge Network, Wanfang Data Knowledge Service Platform, VIP Chinese Periodical Service Platform, and China Biomedical Literature Database for systematic reviews and meta-analyses that investigated the association between dietary fiber and risk of cancer incidence. Keywords and free text search terms were obtained from MeSH and Emtree databases. Search terms: “Dietary Fiber”, “Dietary Fibers”, “Wheat Bran”, “Wheat Brans”, “Roughage”, “Roughages”, “Soluble dietary fiber”, “Insoluble dietary fiber”, “Fiber”, “Hemicellulose”, “Lignin”, “Pectin”, “Neoplasms”, “Tumor”, “Neoplasm”, “Tumors”, “Neoplasia”, “Neoplasias”, “Cancer”, “Cancers”, “Malignant Neoplasm”, “Malignancy”, “Malignancies”, “Malignant Neo-plasms”, “Meta-Analysis as Topic”, “Meta-Analysis”, “Systematic Review”, “meta-analysis”, “data pooling”, “data poolings”, “clinical trial overview”, and “clinical trial overviews”. The detailed PubMed search strategy is shown in Table 1, and other databases are adjusted based on the PubMed search strategy. In addition, we hand-searched the reference lists of the eligible studies.

### 2.2. Inclusion and Exclusion Criteria

The inclusion criteria of the study were as follows: meta-analysis or systematic reviews published in English or Chinese that investigated the association between dietary fiber and cancer incidence, and reported effect size (ES) as well as a 95% confidence interval (CI).

The exclusion criteria of the study were as follows: meta-analysis or systematic reviews of any nutritional and dietary pattern interventions or single fiber intake, narrative reviews or general reviews, and animal models as well as other basic research were excluded. When multiple meta-analyses examined the same cancer, we chose the latest meta-analysis with the largest number of studies. In addition, books, editorials, notes, and letters were also excluded.

After a preliminary search, all the retrieved literature was imported into EndNote 20 and only one duplicate of the literature was retained. Following the established inclusion and exclusion criteria, the retrieved literature underwent initial screening, after which the remaining literature was independently reviewed and screened by two researchers (J.H. and J.W.). Any discrepancies in the results were resolved through discussion with a third researcher (K.X.).

### 2.3. Data Extraction

The following information was extracted from the final selected literature: the first author’s name, year of publication, type, and number of studies included, number of cases and sample size, exposure (dietary fiber type), outcome (cancer site), corresponding ES, and its 95% CI, dose–response relationship (if any), effect model, *p* or *I*^2^ values for heterogeneity testing in a meta-analysis, publication bias, methodology assessment tools and adjustment of covariates and stratification. All data extraction was independently completed by two researchers (J.H. and J.W.), and the differences were resolved through discussion with a third researcher (K.X.).

### 2.4. Methodology Quality

AMSTAR-2 [12] was used to evaluate the methodological quality of all included documents. AMSTAR-2 is a verification tool for systematic reviews suitable for non-randomized intervention studies, with good consistency, reliability, and feasibility. It consists of 16 items, which are divided into key items and non-key items. The key items are item 2, item 4, item 7, item 9, item 11, item 13 and item 15. Based on this, the methodological quality of the study is divided into four levels: (1) high: no or one non-critical weakness; (2) moderate: more than one non-critical weakness; (3) low: one critical flaw with or without non-critical weaknesses; and (4) critically low: more than one critical flaw with or without non-critical weaknesses.

### 2.5. Evidence Quality Assessment

Evidence was graded according to the criteria in the WCRF/AICR expert report [13]. The grading system has five tiers of evidence: (1) convincing evidence: meta-analyses of prospective cohort studies with evidence of dose–response relation, no heterogeneity, no potential confounding factors identified, and eventual disagreement of results over time was reasonably explained. (2) Probable evidence: when there was a high number of studies for meta-analysis (at least 6 case–control or 3 prospective cohort studies), number of cases (>1000+ cases), and no high or unexplained heterogeneity (*I*^2^ < 75%), no potential confounders were identified and the eventual divergence of results over time was reasonably explained. (3) Suggestive evidence: when meta-analyses of prospective cohort studies existed with missing information on significant heterogeneity and identification of potential confounders (such as different findings in subgroups). (4) Inconclusive evidence: when only meta-analyses of case–control studies, or limited prospective cohort studies in meta-analyses (*n* < 3), or when there are significant differences in the results of meta-analyses of the same level of evidence. (5) Improbable evidence: when there were meta-analyses of prospective studies with no significant statistical association, non-significant dose–response association, a large number of studies or cases, no high and unexplained heterogeneity (*I*^2^ < 75%), the robustness of results in sensitivity analyses and no plausible mechanisms. Finally, convincing, probable, and improbable evidence is considered strong, while suggestive or inconclusive evidence is considered limited or undesirable.

### 2.6. Data Analysis

The purpose of the umbrella evaluation was to provide a comprehensive assessment of existing meta-analyses for a specific question instead of repeating the search for original articles for meta-analysis [14]. Therefore, only the ES of the association between dietary fiber and cancer along with its 95% CI were extracted for each meta-analysis included, rather than searching the original studies and re-running the meta-analysis. Heterogeneity was assessed based on the *p* value or *I*^2^ value of the heterogeneity analysis. Significant heterogeneity was considered to exist when the heterogeneity *p* < 0.1 or *I*^2^ ≥ 75.0%. Publication bias of meta-analysis was assessed by Egger’s test, Begg’s test, or funnel plot, and publication bias could be considered when the *p* value was less than 0.1.

## 3. Results

### 3.1. Research Screening

**Figure 1:**
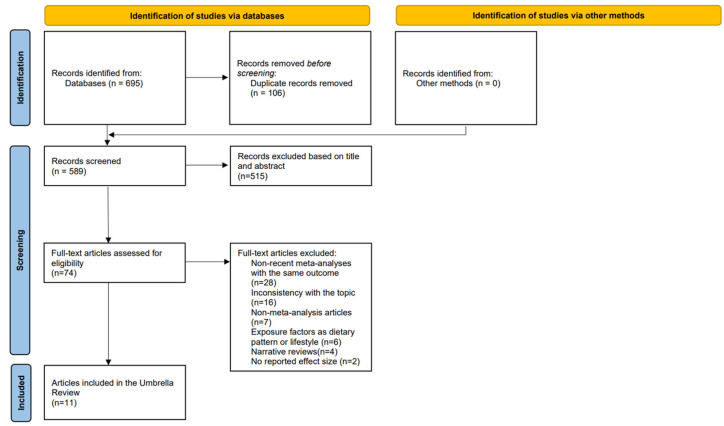
Flow diagram of the enrollment of systematic reviews/meta-analyses of the association of dietary fiber with cancer.

A total of 695 relevant studies were retrieved from PubMed, Web of Science, and other databases, with 589 articles remaining after excluding duplicate articles (*n* = 106). Subsequently, 74 articles remained after a primary screening by title and abstract. The remaining 74 articles were further excluded by reading the full text for reasons including non-recent meta-analyses with the same outcome (*n* = 28), inconsistency with the topic (*n* = 16), non-meta-analysis articles (*n* = 7), exposure factors such as a dietary pattern or lifestyle (*n* = 5), narrative reviews (*n* = 4), and no reported effect size (*n* = 2). Ultimately, 11 articles exploring the association between dietary fiber and cancer were included in the study [15,16,17,18,19,20,21,22,23,24,25]. Figure 1 illustrates the literature screening process. A detailed inclusion–exclusion list can be found in Supplementary Material Table S1.

### 3.2. The Characteristics of the Included Study

A total of 11 meta-analyses, published from 2013–2021, included 1 article in Chinese and 10 articles in English. All articles included a variable number of case–control studies and prospective cohort studies, except for the meta-analysis examining dietary fiber and esophageal cancer, which included only case–control studies. Seven articles used assessment tools such as NOS (The Newcastle Ottawa Scale) and ROB (the basis of The Cochrane Collaboration Recommendations), while the remaining four articles did not report methodological assessment tools. The included meta-analysis reported the association between dietary fiber and 11 cancers. A detailed description is shown in Table 2.

### 3.3. Methodology Quality Assessment

The methodological quality of the included meta-analyses was assessed by applying the AMSTAR-2 scale. The overall quality of the studies was low, with only one meta-analysis classified as a high-quality study, seven as low-quality studies, and three as very low-quality studies. The reason why is that most of the studies did not pro-vide a description of the selection of the included study types and did not provide a list of excluded studies and ex-plain the reasons for exclusion. The AMSTAR-2 scale scores for the 11 meta-analyses of included studies were shown in Supplementary Material Table S2.

### 3.4. Evidence Quality Evaluation

**Figure 2:**
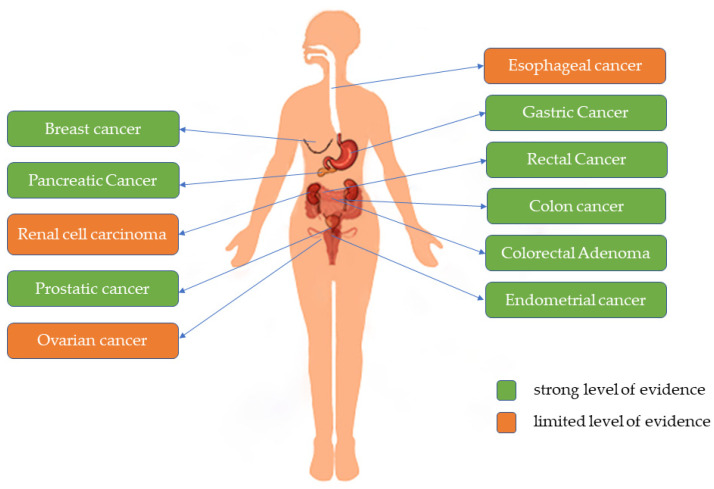
Level of evidence for the association between dietary fiber and cancer.

The quality of evidence for the outcome of the included meta-analysis was evaluated by the criteria in the WCRF/AICR expert report, involving a total of 11 cancers. The results of grading showed that the protective effect of dietary fiber on gastric cancer was convincing evidence. The protective effect of dietary fiber on rectal, colon, breast, endometrial, pancreatic, colorectal adenoma, and prostate cancers was probable evidence, on ovarian and renal cell cancers was suggestive evidence, while on esophageal cancers was inconclusive evidence. The evidence for a total of 8 cancers can be considered strong according to the criteria, and the rest of the evidence levels were decreased mostly due to the absence of dose-response analysis and significant heterogeneity or only meta-analysis of case-control studies. The criteria for evaluating the quality of evidence and the results were shown in Table 3. Figure 2 demonstrated the level of evidence for the protective effect of dietary fiber on the risk of various cancers. The detailed evaluation data table was shown in Supplementary Material Table S3.

### 3.5. Association Analysis of Dietary Fiber and Cancer

#### 3.5.1. Esophageal and Gastric Cancer

Lingli Sun investigated the influence of dietary fiber on esophageal cancer in a case–control study based on both population and hospital cases. The study yielded inconclusive evidence that dietary fiber intake could reduce the risk of esophageal cancer (OR = 0.52; 95% CI = 0.43–0.64), and further dose–response analysis yielded a 31% reduction of the risk of esophageal cancer with each 10 g/d increase in dietary fiber intake (OR = 0.69; 95% CI = 0.61–0.79). Despite the fact that dietary fiber is undigested in the stomach after entering it from the esophagus, there is strong evidence that it has a protective effect against gastric cancer (OR = 0.58; 95% CI = 0.49–0.67). Moreover, each 10 g/d increase in dietary fiber intake led to a 44% decrease in the risk of gastric cancer (OR = 0.56; 95% CI = 0.45–0.71).

#### 3.5.2. Colon and Rectal Cancers and Colorectal Adenomas

Dietary fiber, upon entering the intestine from the stomach, was broken down and fermented by specific gut microbiota, which contributed to improving gut health. In contrast to previous studies that combined the effects of dietary fiber on colorectal cancer, Vincenza Gianfredi’s team conducted separate meta-analyses for colon and rectal cancers. The results showed probable evidence that dietary fiber intake reduced the risk of colon cancer (ES = 0.74; 95% CI = 0.67–0.82) and rectal cancer (ES = 0.77; 95% CI = 0.66–0.89). Given the incurability of colorectal cancer, Daniele Nucci conducted research on colorectal adenoma, a precancerous lesion of colorectal cancer, and there was probable evidence of a protective effect of dietary fiber intake on the development of colorectal adenoma (ES = 0.71; 95% CI = 0.68–0.75).

#### 3.5.3. Breast, Ovarian, and Endometrial Cancer

Dietary fiber played a protective role against certain types of cancers of the esophagus and gastrointestinal tract, as well as some female malignant neoplasms. A meta-analysis of prospective studies showed probable evidence that increased dietary fiber intake likely offered protection against breast cancer (RR = 0.92; 95% CI = 0.88–0.95). Subgroup analyses considering important influencing factors such as menopausal status revealed that higher total fiber intake correlated with a lower risk of both pre- and post-menopausal breast cancer (RR = 0.82; 95% CI = 0.67–0.99), (RR = 0.91; 95% CI = 0.88–0.95). Moreover, the article conducted a detailed analysis of the type of dietary fiber and found a significant negative association between soluble dietary fiber and breast cancer risk (RR = 0.90; 95% CI = 0.84–0.96), as well as a significant negative association between insoluble fiber and the risk of breast cancer (RR = 0.93; 95% CI = 0.86–1.00).

In addition, there was probable evidence that dietary fiber intake also played a protective role against endometrial cancer (RR = 0.86; 95% CI = 0.78–0.93). In another female malignancy, ovarian cancer, research showed a negative association between total dietary fiber intake and risk of ovarian cancer (RR = 0.70; 95% CI = 0.57–0.87) as suggestive evidence, and dose–response analysis found a 3% reduction of risk of ovarian cancer for each 5 g/d increase in dietary fiber intake (RR = 0.97; 95% CI = 0.95–0.99).

#### 3.5.4. Other Cancers

Dietary fiber was also found to be protective against prostate cancer, renal cell carcinoma, and pancreatic cancer, with effect sizes of (RR = 0.87; 95% CI = 0.77–0.99), (RR = 0.84; 95% CI = 0.74–0.96), and (ES = 0.63; 95% CI = 0.53–0.76), respectively, where the protective effect of dietary fiber on prostate cancer and pancreatic cancer was probable evidence and on renal cell carcinoma was suggestive evidence. The results of subgroup analysis showed that both soluble and insoluble dietary fiber were protective against prostate cancer (RR = 0.78; 95% CI = 0.64–0.95), (RR = 0.65; 95% CI = 0.45–0.88), but the dose–response relationship did not show a significant association. Dietary fiber from vegetable and legume sources was protective against renal cell carcinogenesis (RR = 0.70; 95% CI = 0.49–1.00), (RR = 0.80; 95% CI = 0.69–0.93), but there was no statistically significant association for dietary fiber from fruit and grain sources, and no dose–response relationship.

## 4. Discussion

This study explored the association between dietary fiber and cancer through an umbrella review of 11 meta-analyses. In this article, the methodological quality was assessed using the AMSTAR-2 scale, and the quality of evidence was graded using the criteria in the WCRF/AICR expert report. We found that the overall level of methodological quality of the included meta-analysis was suboptimal, but the level of evidence quality was persuasive. Moreover, the study results showed that those with higher dietary fiber intake had a lower risk of several types of cancer compared to the lower, which indicates that dietary fiber has a preventive effect on cancer to a certain extent.

The preventive effect of dietary fiber on the development of cancer is consistent with the results of a meta-analysis of a large sample published by Andrew Reynolds in *The Lancet*, which showed a 13% reduction of cancer mortality in those who consumed the most dietary fiber compared to those who ate the least [26]. It is important to note, however, that excessive intake of dietary fiber may lead to side effects such as flatulence, bloating, loose stools or diarrhea, and abdominal cramps [27].

According to a recent report by the World Health Organization, colorectal cancer is the third most common type of cancer worldwide, with almost 20,000 cases diagnosed in 2020 alone. In addition, colorectal cancer was the second most common cause of cancer death, causing nearly 10,000 deaths each year. Despite the availability of effective screening techniques, the situation remains bleak [28]. Colorectal adenoma, a potential precancerous lesion of colorectal cancer, may share the same pathogenesis as colorectal cancer. Dietary fiber can increase stool volume and decrease stool transit time, thus diluting the concentration of carcinogens in the colon and reducing the time of intestinal exposure to carcinogens [29]. Indeed, dietary fiber binds bile acids and alters the enterohepatic axis, which can reduce cholesterol levels involved in the etiology of colon cancer [30]. In contrast, secondary bile acids produced by bile acid metabolism are thought to be promoters of colorectal cancer [31,32], which can cause significant damage to the colonic mucosa, such as oxidative stress and inflammation [33]. Dietary fiber is broken down by intestinal flora into short-chain fatty acids (SCFA), such as acetate, propionate, and butyrate, which can decrease intestinal luminal pH, which helps reduce the conversion of proto-bile acids to carcinogenic secondary bile acids [34]. Moreover, butyrate not only provides energy to normal colonic epithelial cells but also induces excessive activation of Wnt signaling in colon cancer cells, an event necessary to achieve high levels of apoptosis in these cells, where Wnt activity is associated with cancer cell proliferation [35]. Furthermore, under acidic conditions, dietary fiber has been shown to remove nitrite from the stomach [36] and decrease the concentration of nitroso compounds, which increase the risk of gastric cancer [37], whereas esophageal cancer can be divided into esophageal squamous carcinoma and esophageal adenocarcinoma. It has been shown that dietary fiber is negatively associated with precancerous lesions in esophageal cancer, and the potential mechanisms include improvement of gastroesophageal reflux and weight control, adsorption of carcinogens contained in food, improvement of cancer-associated esophageal ecological dysregulation, and direct action on cancer cells [23,38,39,40].

According to the latest reports from the World Health Organization, breast cancer has become the most prevalent form of cancer worldwide in 2020. There were over 20,000 new cases of breast cancer and nearly 266,850 deaths from the disease globally. Breast cancer is now the leading cause of cancer-related deaths in women and the fifth most common cause of cancer death overall [28]. Estrogen stimulation is thought to play a causal role in the pathogenesis of breast cancer [41]. In human studies, there is a consistent relationship between serum estrogen concentrations and breast cancer risk [42,43]. A combined analysis of prospective studies found a highly significant positive association between serum bioavailable estradiol concentrations and breast cancer risk in postmenopausal women [43]. In contrast, dietary fiber may decrease circulating estrogen concentrations by inhibiting bacterial β-glucosidase activity in the intestine, thereby inhibiting estrogen reabsorption in the colon and increasing fecal estrogen excretion, thereby reducing the risk of breast cancer [44]. Disruption of the enterohepatic circulation of cholesterol (a precursor of estrogen) may as well decrease the risk of breast cancer [30]. Maskarinec G et al. showed that a diet high in dietary fiber increased sensitivity to insulin and decreased the levels of estrogens such as estradiol and estriol, thus preventing breast cancer [45]. Finally, natural active phytochemicals coexisting with dietary fiber natural compounds in dietary fiber (such as phytoestrogens, phenolic acids, and lignans) play an important protective role against breast cancer through their antioxidant properties and their ability to inhibit cell proliferation and angiogenesis and induce apoptosis, together with through modulation of hormonal pathways [46]. Since estrogen stimulation is also a strong causative factor for endometrial cancer [41], the estrogen-inhibiting and pro-excretory effects of dietary fiber play a protective role against endometrial carcinogenesis. Alternatively, dietary fiber intake has been shown to lower blood pressure levels [47] and reduce the risk of diabetes [48,49], both of which are risk factors for endometrial cancer. Therefore, there is strong indirect evidence to support the negative association between dietary fiber intake and endometrial cancer risk.

Various mechanisms have been proposed to elucidate the protective effect of dietary fiber intake on ovarian cancer. Dietary fiber may hinder the progression of ovarian cancer by modifying bacterial macroflora and enhancing excretion, resulting in reduced serum levels and availability of estrogens, and ultimately reducing the bioavailability of steroid hormones [50,51,52]. In addition to estrogen-related pathways, dietary fiber is thought to reduce glycemic load and improve insulin sensitivity, thereby affecting insulin-like growth factor (IGF), which is considered a risk factor for ovarian cancer [53,54]. Meanwhile, it has been found that insulin resistance and hyperinsulinemia may stimulate prostate cancer development by decreasing IGF binding protein and increasing free IGF concentration [55]. In contrast, dietary fiber may reduce prostate cancer risk by decreasing carbohydrate absorption to reduce insulin resistance [56] and improving insulin sensitivity [55].

There are two hypotheses regarding the protective effect of dietary fiber on pancreatic cancer. First, dietary fiber intake can influence insulin insensitivity or insulin resistance pathways [57,58], which are associated with the etiology of pancreatic cancer [59]. The second hypothesis is that dietary fiber may have an effective protective effect due to its anti-inflammatory properties [60]. Some studies have shown that chronic pancreatitis is a risk factor for pancreatic cancer [61,62]. Experimental studies have demonstrated that dietary fiber has a down-regulatory effect on the transcription of genes related to inflammatory factors and protein expression [63], possibly through the anti-inflammatory properties of fermentation products, particularly butyrate [64]. The anti-inflammatory properties of butyrate are also one of the reasons why dietary fiber plays a protective role against renal cell carcinoma, which is additionally known to be one of the malignancies associated with obesity, with a positive correlation between the risk of its development and increased Body Mass Index (BMI) [65]. Dietary fiber may promote satiety and weight loss by increasing stool volume and stool transit time [66]. Moreover, dietary fiber intake may reduce the risk of morbidity. In addition, dietary fiber may also control postprandial blood glucose by slowing the entry of glucose from the intestine into the bloodstream [67]. The avoidance of developing diabetes can reduce the risk of developing pancreatic cancer [68].

The advantage of this study is that it has comprehensively evaluated the protective effect of dietary fiber on various cancers. Compared with digestive tract tumors that have received more attention, people should also pay attention to the preventive effect of dietary fiber on high-incidence malignant female tumors such as breast cancer and endometrial cancer. Moreover, this study also comprehensively analyzed the relationship between pancreatic cancer, prostate cancer as well as renal cell cancer and dietary fiber. Of course, this study also has some limitations. Firstly, the inclusion of meta-analyses cannot avoid the existence of confounding factors. Different survey populations and different survey methods all have an impact on the results. Nonetheless, most of the meta-analyses included in this study have conducted sensitivity analysis and hierarchical analysis, which can reduce the impact of confounding factors on the results. Secondly, the calculation of dietary fiber intake in the included studies may be inaccurate due to the wide variety and inconsistent definition of dietary fiber. To avoid heterogeneity, this study uses total dietary fiber as an exposure factor. Finally, since most of the included studies are observational, the causal relationship between dietary fiber intake and tumorigenesis cannot be established.

## 5. Conclusions

In summary, our study reveals that dietary fiber offers protective effects against various types of malignancies, including gastrointestinal cancers (such as colorectal, gastric, and esophageal cancers) and female-specific cancers (such as breast, endometrial, and ovarian cancers), as well as pancreatic, prostate, and renal cell cancers. The study provides strong evidence for the protective effects of dietary fiber on most cancers, indicating that increasing the intake of dietary fiber is crucial for cancer prevention and promoting population health. Therefore, clinicians should consider recommending an increase in dietary fiber intake while providing nutritional guidance to the population. Nonetheless, we must exercise caution when assessing studies with low-quality evidence.

In view of the progress of today’s food science and technology, dietary fiber supplement products have come out one after another. Future research can focus on whether the presence of dietary fiber supplements can fill the serious shortage of dietary fiber in people today. Further exploration is required to identify the optimal dietary fiber composition for dietary supplements and determine if they can replace or supplement the role of dietary fiber in conventional foods.

## Figures and Tables

**Table 1:** Strategy for searching systematic reviews/meta-analyses of the association of dietary fiber with cancer in PubMed.

Step	Search Strategy
#1	(“Dietary Fiber” [Mesh] OR Dietary Fibers [Title/Abstract] OR Wheat Bran [Title/Abstract] OR Wheat Brans [Title/Abstract] OR Roughage [Title/Abstract] OR Roughages [Title/Abstract] OR Soluble dietary fiber [Title/Abstract] OR Insoluble dietary fiber [Title/Abstract] OR Fiber [Title/Abstract] OR Hemicellulose [Title/Abstract] OR Lignin [Title/Abstract] OR Pectin [Title/Abstract]) AND (“Neoplasms” [Mesh] OR Tumor [Title/Abstract] OR Neoplasm [Title/Abstract] OR Tumors [Title/Abstract] OR Neoplasia [Title/Abstract] OR Neoplasias [Title/Abstract] OR Cancer [Title/Abstract] OR Cancers [Title/Abstract] OR Malignant Neoplasm [Title/Abstract] OR Malignancy [Title/Abstract] OR Malignancies [Title/Abstract] OR Malignant Neoplasms [Title/Abstract])
#2	“Meta-Analysis as Topic” [Mesh] OR “Meta-Analysis” [Publication Type] OR “Systematic Review” [Publication Type] OR meta-analysis [Title/Abstract] OR data pooling [Title/Abstract] OR data poolings [Title/Abstract] OR clinical trial overview [Title/Abstract] OR clinical trial overviews [Title/Abstract]
#3	#1 AND #2

**Table 2:** Basic characteristics of included meta-analysis.

Cancer	Number of Studies	Number and Type of Study Included	Number of Cases/Sample Size	Assessed with	Main Result	Heterogeneity	Dose–Response	Publication Bias	Methodology Assessment Tools
Prostatic cancer	10	5C, 5CC	12,058/254,213	TDF, IDF, SDF, FDF, VDF, CDF	RR (TDF): 0.87 (0.77–0.99); RR (IDF): 0.65 (0.45–0.88); RR (SDF): 0.78 (0.64–0.95); RR (FDF): 0.98 (0.85–1.12); RR (VDF): 0.91 (0.70–1.18); RR (CDF): 1.03 (0.95–1.12)	TDF: *I*^2^ = 56.6%; IDF: *I*^2^ = 56.0%; SDF: *I*^2^ = 0.0%; FDF: *I*^2^ = 31.4%; VDF: *I*^2^ = 75.0%; GDF: *I*^2^ = 0.0%	Per 1 g/day increment in total dietary fiber RR: 0.996 (0.989–1.002)	*p* = 0.064	NOS
Breast cancer	20	2CC, 17C, 1CT	69,735/2,092,037	TDF, IDF, SDF, FDF, VDF, CDF, LDF	RR (TDF): 0.92 (0.88–0.95); RR (IDF): 0.93 (0.86–1.00); RR (SDF): 0.90 (0.84–0.96); RR (FDF): 0.93 (0.89–0.96); RR (VDF): 0.95 (0.90–1.00); RR (CDF): 0.97 (0.93–1.01); RR (LDF): 0.97 (0.92–1.03)	TDF: *I*^2^ = 12.6%; IDF: *I*^2^ = 33.4%; SDF: *I*^2^ = 12.6%; FDF: *I*^2^ = 9.0%; VDF: *I*^2^ = 39.6%; CDF: *I*^2^ = 29.6%; LDF: *I*^2^ = 0.0%		*p* > 0.05	NR
Rectal cancer	22	9CC, 13C	>1000/2,876,136	TDF	RR: 0.77 (0.66–0.89)	*I*^2^ = 9.1%		*p* = 0.816	ROB
Colon cancer	21	8CC, 13C	>1000/2,627,391	TDF	RR: 0.74 (0.67–0.82)	*I*^2^ = 43.8%		*p* = 0.177	ROB
Renal cell carcinoma	7	4CC, 3C	6115/941,202	TDF, IDF, SDF, FDF, VDF, CDF, LDF	RR (TDF): 0.84 (0.74–0.96); RR (FDF): 0.92 (0.80–1.05); RR (VDF): 0.70 (0.49–1.00); RR (CDF): 1.04 (0.91–1.18); RR (LDF): 0.80 (0.69–0.93)	TDF: *I*^2^ = 23.8%; FDF: *I*^2^ = 0.0%; VDF: *I*^2^ = 76.9%; GDF: *I*^2^ = 0.0%; LDF: NA	Per 10 g/day increment in total dietary fiber RR: 0.94 (0.80–1.11)	Egger’s test and Begg’s test (*p*= 0.728, *p*= 0.707, respectively)	NOS
Endometrial cancer	16	13CC, 3C	6563/198,174	TDF	RR: 0.86 (0.78–0.93)	*I*^2^ = 69.1%		*p* > 0.05	NOS
Pancreatic cancer	18	15CC, 3C	>1000/343,120	TDF	RR: 0.63 (0.53–0.76)	*I*^2^ = 68.2%		*p* = 0.006	NR
Colorectal adenoma	21	12CC, 8C, 1CT	>1000/157,725	TDF	RR: 0.71 (0.68–0.75)	*I*^2^ = 62.7%		*p* = 0.838	NR
Esophageal cancer	15	15CC	3625/16,885	TDF	RR: 0.52 (0.43–0.64)	*I*^2^ = 71.6%	Per 10 g/day increment in total dietary fiber RR: 0.69 (0.61–0.79)	*p* > 0.05	NOS
Ovarian cancer	19	14CC, 5C	8200/567,742	TDF	RR: 0.70 (0.57–0.87)	*I*^2^ = 83.5%		*p* = 0.276	NR
Gastric cancer	21	19CC, 2C	6950/580,064	TDF	RR: 0.58 (0.49–0.67)	*I*^2^ = 62.2%	Per 10 g/day increment in total dietary fiber RR: 0.56 (0.45–0.71)	*p* = 0.931	NOS

Abbreviation: C: cohort study; CC: case–control study; CT: clinic trial; TDF: total dietary fiber; SDF: soluble dietary fiber; IDF: insoluble dietary fiber; VDF: vegetable dietary fiber; FDF: fruit dietary fiber; CDF: cereal dietary fiber; LDF: legumes dietary fiber; NR: not referenced.

**Table 3:** Evidence quality level evaluation criteria and results.

Level of Evidence ^a^	Criteria ^b^	Cancer
Convincing	Meta-analyses of prospective cohort studies with evidence of dose–response relation, no heterogeneity, no potential confounding factors identified, and eventual disagreement of results over time reasonably explained.	Gastric cancer
Probable	Meta-analyses should include at least six case–control or three prospective cohort studies, with a sample size greater than 1000 cases, no high or unexplained heterogeneity (*I*^2^ < 75%), no identified potential confounders, and the final difference in results over time was reasonably explained.	Endometrial cancer Rectal cancer Colon cancer Breast cancer Prostatic cancer Pancreatic cancer Colorectal adenoma
Suggestive	Meta-analyses of prospective cohort studies with a lack of information on significant heterogeneity (*I*^2^ < 75%) and identification of potential confounding factors (such as different findings in subgroups).	Ovarian cancer Renal cell carcinoma
Inconclusive	Meta-analyses of case–control studies, limited prospective cohort studies included in meta-analyses (*n* < 3), or evident contrasting results from meta-analyses with the same level of evidence.	Esophageal cancer
Improbable evidence	Meta-analyses of prospective cohort studies with no significant statistical association, non-significant dose–response association, a large number of studies or cases, no high and unexplained heterogeneity (*I*^2^ < 75%), the robustness of results in sensitivity analyses, and no plausible mechanisms.	None

^a^ The convincing, probable, and improbable evidence was considered strong, and suggestive or not conclusive evidence was considered limited or poor. Green indicates a strong level of evidence, and orange indicates a limited level of evidence. ^b^ The criteria from the WCRF/AICR Expert Report were adopted.

## Data Availability

Reported data from the systematic reviews incorporated in the present umbrella review can be found in the original systematic reviews cited in the References section.

## Supplementary Materials

The following supporting information can be downloaded at: https://www.mdpi.com/article/10.3390/nu15112545/s1, Table S1: Literature screening details and exclusion reasons. Table S2: Methodological quality evaluation of literature. Table S3: Evaluation of evidence grade.
