# Supplementary material for: Use of Dietary Fibers in Reducing the Risk of Several Cancer Types: An Umbrella Review
Source: Nutrients. 2023 May 30;15(11):2545. doi: 10.3390/nu15112545 (PMC10255454; doi:10.3390/nu15112545)
Supplement: Supplementary file 1 [file nutrients-15-02545-s001.zip › nutrients-2360385-supplementary.pdf]

Table S1: Literature screening details and exclusion reasons

First author	Year of publication	Regional distribution (number of studies)	Number of included studies						Sample size	Types of dietary fiber	Outcome	Inclusion	Reasons for exclusion
			Case-control studies	Cohort studies	RCT	clinical trial	Observational studies	total					
Kun Chen	2002	China（14）	14	0	0	0	0	14	10,239	TDF	Colorectal cancer	No	Same studies as Vincenza Gianfredi et al.
Weishuai Liu	2016	Asia（2），Europe（4），United States（4）	5	5	0	0	0	10	254,213	TDF, SDF, IDF, CDF、FDF, VDF	prostate cancer	Yes	
Zuwang Liu	2015	China（1），Europe（4），United States（11）	0	16	0	0	0	16	475,937	TDF	breast cancer	No	Same studies as Maryam S. Farvid et al.
Hongmei Shao	2014	China（25）	25	0	0	0	0	25	16,603	Coarse grains, vegetables, fruits	Colon cancer	No	no effect size
Ming Xu	2015											No	narrative review
Dang Zhang	2017	NA	15	0	0	0	0	15	1,092,863	TDF	breast cancer	No	Same studies as Maryam S. Farvid et al.
Wei Zhang	2015											No	narrative review

Qian Zhou	2019	China（9），Europe（2）	11	0	0	0	0	11	10,890	Low dietary fiber intake	Colon cancer	No	Same studies as Daniele Nucci et al.
Mehmet Emin Arayici	2022	Asia（5），United States（3），Europe（3）	7	5	0	0	0	12	433,162	TDF, SDF, IDF	Colorectal cancer	No	Same studies as Vincenza Gianfredi et al.
Tracey K. Asano	2002	United States（2），Europe（3）	0	0	5	0	0	5	4,349	Integrated meals	Colorectal adenoma	No	Same studies as Daniele Nucci et al.
D. Aune	2012	United States（9），Europe（6），Asia（1）	0	16	0	0	0	16	NA	TDF, FDF, VDF, CDF, SDF, IDF	breast cancer	No	Same studies as Maryam S. Farvid et al.
Dagfinn Aune research associate	2011	United States（12），Europe（5），Asia（4）	0	21	0	0	0	21	1,985,552	TDF, FDF, VDF, LDF，CDF, Whole grains	Colon cancer	No	Same studies as Vincenza Gianfredi et al.
Dagfinn Aune	2016	Europe（20），United States（16），Asia（9）	0	45	0	0	0	45	640,065	Whole grain fiber	Total cancer	No	beside the topic
Elisa V Bandera	2007	China（2），North America（8）	9	1	0	0	0	10	13,069	TDF	Endometrial cancer	No	Same studies as Hengjie Li et al.

Nerea Becerra-Tomás	2021	NA	0	0	4	0	104	108	>15,100	TDF	All-cause mortality	No	beside the topic
Qiwen Ben	2014	United States（12）, Europe（6）, Asia（2）	16	4	0	0	0	20	10,948	TDF, FDF, VDF, CDF	Colorectal adenoma	No	Same studies as Daniele Nucci et al.
Sanaz Benisi-Kohansal	2016	United States（11）, Europe（8） China（1）	0	20	0	0	0	20	2,282,603	Whole grain fiber	Total cancer mortality, stomach cancer, lung cancer, colon cancer	No	beside the topic
Guo-Chong Chen	2016	United States（6）, Europe（7）, Asia（1）	0	14	0	0	0	14	NA	Whole grain fiber	All-cause mortality	No	beside the topic
Kangning Chen	2018	North America（11）, Europe（2）, China（2）	12	3	0	0	0	15	110,374	TDF, CDF, VDF, FDF, IDF	Endometrial cancer	No	Same studies as Hengjie Li et al.
Sumei Chen	2016	North America（10）, Europe（12）, Asia（2）	0	0	0	0	0	24	3,662,421	TDF	breast cancer	No	Same studies as Maryam S. Farvid et al.
Helen G Coleman	2013	United States（6）, Europe	10	0	0	0	0	10	10,436	TDF	Adenocarcinoma of the esoph	No	Same studies as Lingli Sun et al.

		（3），Asia（1）									agus, squamous cell carcinoma of the esophagus		
Christina C. Dahm	2010	United Kingdom（7）	0	7	0	0	0	7	2,575	Dietary fiber density, TDF	colorectal cancer	No	Same studies as Vincenza Gianfredi et al.
Jia-Yi Dong	2011	North America（5），Europe（4），China（1）	0	10	0	0	0	10	712,195	TDF	breast cancer	No	Same studies as Maryam S. Farvid et al.
Maryam S. Farvid	2020	North America（10），Europe（8），Australia（1），Asia（2）	2	17	0	2	0	21	2,092,037	TDF, CDF, FDF, VDF, LDF	breast cancer	Yes	
Glenn A. Gaesser	2020	NA										No	beside the topic
Vincenza Gianfredi	2019	Europe（8），United States（4），Asia（6）	6	12	0	0	0	18	2,176,814	TDF	Rectal cancer	Yes	
Vincenza Gianfredi	2018	Europe（8），North America（6），Asia（7）	14	7	0	0	0	21	3,742,355	TDF	Colon cancer	Yes	

P. HAAS	2009	NA	0	11	0	0	0	11	1,511,171	TDF	colorectal cancer	No	no effect size
Maryam Hajishafiee	2016	United States（10），Europe（5），Australia（2），Asia（1）	0	18	0	0	0	18	1,740,040	CDF	Total cancer mortality	No	beside the topic
Tung Hoang	2020											No	dietary pattern and lifestyle
Tian-bao Huang	2014	North America（4），Europe（3）	5	2	0	0	0	7	941,202	TDF, CDF, FDF, VDF, LDF	Renal cell carcinoma	Yes	
Xiumin Huang	2018	United States（10），Europe（4），Asia（3）	13	4	0	0	0	17	149,117	TDF	Risk of ovarian cancer	No	Same studies as Hui Xu et al.
Masoumeh Jabbari	2022											No	review without meta-analysis
Elizabeth T Jacobs	2006											No	review without meta-analysis
Ahmad Jayedi	2020	North America（5），Europe（1），Australia（1）	0	7	0	0	0	7	11,295	TDF	breast cancer mortality	No	Same studies as Maryam S. Farvid et al.
Alireza Khodavandi	2020											No	dietary pattern and lifestyle

Youngyo Kim	2015	Europe（7），United States（5），Australia（2），Asia（1）	0	15	0	0	0	15	1,409,014	TDF	Total cancer mortality	Yes	
Ai Seon Kuan	2019											No	review without meta-analysis
Qiucheng Lei	2016	Europe（3），United States（5）	7	1	0	0	0	8	43,629	TDF	pancreatic cancer	No	Same studies as Daniele Nucci et al.
Hengjie Li	2019	United States（10）Europe（4），Asia（2）	13	3	0	0	0	16	198,174	TDF	Endometrial cancer	Yes	
Ning Li	2022	Asia（3），Australia（3），Europe（22），North America（23）	0	51	0	0	0	51	2,725,657	TDF	Breast cancer incidence and mortality	No	beside the topic
Lihua Liu	2014	United States（13），Europe（10），Asia（1），NA（1）	0	25	0	0	0	25	1,752,848	TDF	Total cancer mortality	No	Same studies as Youngyo Kim et al.

Yu Ma, MD	2018	United States（7），Europe（4）	0	11	0	0	0	11	1,450,553	TDF	Proximal colon cancer, distal colon cancer	No	Same studies as Vincenza Gianfredi et al.
Qi-qi Mao	2017	North America（6），Europe（5），Asia（2），Australia（1）	13	1	0	0	0	14	38,141	TDF	pancreatic cancer	No	Same studies as Daniele Nucci et al.
Masrul Masrul	2019	Asia（10）	8	2	0	0	0	10	49,964	TDF	Colon cancer	No	Same studies as Vincenza Gianfredi et al.
Marc P. McRae	2018											No	review without meta-analysis
Daniele Nucci	2021	United States（18）Europe（2），Asia（1）	12	8	0	0	1	21	157,725	TDF	Colorectal adenoma	Yes	
Daniele Nucci	2021	Europe（7），North America（7），Asia（2），Australia（1），Multi-center（1）	15	3	0	0	0	18	343,120	TDF	pancreatic cancer	Yes	

Hannah Oh	2019	United States（9），Europe（1）	0	10	0	0	0	10	1,340,841	Whole grain fiber, FDF, VDF, LDF	Colorectal cancer	No	Same studies as Vincenza Gianfredi et al.
Sin-Hye Park	2021	North America（20）Europe（10）Asia（4），Australia（1）							120,167			No	dietary pattern and lifestyle
Yikyung Park	2005											No	review without meta-analysis
Mingyue Rao	2020	Europe（15）United States（1），Australia（4）	0	1	17	2	0	20	NA	Indigestible carbohydrates	Colorectal cancer	No	beside the topic
Andrew Reynolds	2019	NA						NA	4,635	TDF	Colorectal cancer, breast cancer	No	beside the topic
Lukas Schwingshackl	2017	North America（19）Europe（14）Asia（9）Australia（7）	0	0	49	0	0	49	287,304	TDF		No	beside the topic
Tao Sheng	2015	Europe（7），North America（6），South America	12	5	0	0	0	17	255,026	TDF	prostate cancer	No	Same studies as Weishuai Liu et al.

		（1），South Africa（1），Asia（2）											
Lingli Sun	2015	NA	15	0	0	0	0	15	16,885	TDF	Esophageal carcinoma	Yes	
Reiko Suzuki	2009											No	review without meta-analysis
Maria Tieri	2020											No	narrative review
Bruce Trock	1990	NA	23	0	0	0	0	23	NA	TDF	Colon cancer	No	Same studies as Vincenza Gianfredi et al.
Sajesh K. Veettil	2019											No	dietary pattern and lifestyle
Nicola Veronese	2018											No	narrative review
Chun-Hui Wang	2015	North America（6），Europe（5），other（3）	13	1	0	0	0	14	38,111	TDF	pancreatic cancer	No	Same studies as Daniele Nucci et al.
Shurui Wang	2021											No	beside the topic
Yunjun Xiao	2018	Europe（7），United States（2），Asia（2）	7	4	0	0	0	11	131,151	Whole grain fiber	breast cancer	No	beside the topic

Hui Xu	2018	North America（12）Europe（4），Asia（2），Oceania（1）	14	5	0	0	0	19	567,742	TDF	Risk of ovarian cancer	Yes	
Kedi Xu	2022	NA	5	5	0	0	0	10	NA	TDF	breast cancer	No	Same studies as Maryam S. Farvid et al.
Yao Y	2017	NA	0	0	5	0	0	5	4,798	Wheat bran fiber, TDF	Recurrence of colorectal adenoma	No	beside the topic
Evan Y W Yu	2020											No	review without meta-analysis
B Zhang	2017	United States（11）Europe（7），Eastern Mediterranean（1）	0	19	0	0	0	19	1,041,692	Whole grain fiber	Total cancer mortality	No	beside the topic
Xiao-Feng Zhang	2020	United States（15）Europe（15）Asia（4），North Africa（1）	20	15	0	0	0	35	2,663,278	Whole grain fiber	Colorectal cancer, stomach cancer, esophageal cancer	No	beside the topic
Zhizhong Zhang	2013	NA	19	2	0	0	0	21	580,064	TDF	gastric cancer	Yes	
Zhanwei Zhao	2021											No	dietary pattern and lifestyle

Bowen Zheng	2018	North America（9），Europe（2），Australia（2）	10	3	0	0	0	13	142,189	TDF	Ovarian cancer	No	Same studies as Hui Xu et al.
Jiali Zheng	2017											No	dietary pattern and lifestyle

Table S2: Methodological quality evaluation of literature

Author	1	2	3	4	5	6	7	8	9	10	11	12	13	14	15	16	Quality
Liu WS et al [15]	Yes	Yes	No	Partial yes	Yes	Yes	No	Partial yes	Partial yes	No	Yes	Yes	Yes	Yes	Yes	Yes	Low
Maryam S. Farvid [16]	Yes	Yes	Yes	Partial yes	No	No	No	Yes	Partial yes	No	Yes	Yes	Yes	Yes	Yes	Yes	Low
Vincenza Gianfredi [17]	Yes	Yes	No	Partial yes	Yes	Yes	No	Yes	Partial yes	Yes	Yes	Yes	Yes	Yes	Yes	Yes	Low
Vincenza Gianfredi [18]	Yes	Partial yes	No	Partial yes	Yes	Yes	No	Yes	Partial yes	No	Yes	Yes	Yes	Yes	Yes	No	Low
Tian-bao Huang [19]	Yes	No	No	Partial yes	Yes	Yes	No	Yes	Partial yes	No	Yes	Yes	Yes	Yes	Yes	Yes	Very low
Hengjie Li [20]	Yes	Yes	No	Partial yes	No	No	No	Yes	Partial yes	Yes	Yes	Yes	Yes	Yes	Yes	Yes	Low
Daniele Nucci [21]	Yes	Yes	No	Partial yes	Yes	Yes	Partial yes	Partial yes	Partial yes	Yes	Yes	Yes	Yes	Yes	Yes	Yes	High
Daniele Nucci [22]	Yes	Yes	Partial yes	Partial yes	Yes	Yes	Partial yes	Partial yes	Partial yes	Yes	Yes	Yes	Yes	Yes	No	Yes	Low
Lingli Sun [23]	Yes	No	No	Partial yes	Yes	Yes	No	Partial yes	Partial yes	No	Yes	Yes	Yes	Yes	Yes	No	Very low
Hui Xu [24]	Yes	Yes	No	Partial yes	Yes	Yes	No	Partial yes	Partial yes	Yes	Yes	Yes	Yes	Yes	Yes	Yes	Low
Zhizhong Zhang [25]	Yes	No	No	Partial yes	Yes	Yes	No	Partial yes	Partial yes	Yes	Yes	Yes	Yes	Yes	Yes	Yes	Very low

Yes	: the study fully meets the requirements of this item.
Partial yes	: the study partial meets the requirements of this item.
No	: the study does not meet the requirements of this item.

1. Did the research questions and inclusion criteria for the review include the components of PICO? 2. Did the report of the review contain an explicit statement that the review methods were established prior to the conduct of the review and did the report justify any significant deviations from the protocol 3. Did the review authors explain their selection of the study designs for inclusion in the review? 4. Did the review authors use a comprehensive literature search strategy? 5. Did the review authors perform study selection in duplicate? 6. Did the review authors perform data extraction in duplicate? 7. Did the review authors provide a list of excluded studies and justify the exclusions? 8. Did the review authors describe the included studies in adequate detail? 9. Did the review authors use a satisfactory technique for assessing the risk of bias (RoB) in individual studies that were included in the review? 10. Did the review authors report on the sources of funding for the studies included in the review? 11. If meta-analysis was performed did the review authors use appropriate methods for statistical combination of results? 12. If meta-analysis was performed, did the review authors assess the potential impact of RoB in individual studies on the results of the meta-analysis or other evidence synthesis? 13. Did the review authors account for RoB in individual studies when interpreting/ discussing the results of the review? 14. Did the review authors provide a satisfactory explanation for, and discussion of, any heterogeneity observed in the results of the review? 15. If they performed quantitative synthesis did the review authors carry out an adequate investigation of publication bias (small study bias) and discuss its likely impact on the results of the review? 16. Did the review authors report any potential sources of conflict of interest, including any funding they received for conducting the review?

Table S3: Evaluation of evidence grade

cancer	Reference	Number of studies	Number and type of study included	Number of cases/ sample size	Assessed with	Main result	heterogeneity	Dose-response	Publication bias	Plausible confounding	Level of evidence
Prostatic neoplasms	Liu WS [15]	10	5C,5CC	1,2058/ 254,213	TDF, IDF, SDF, FDF, VDF, GDF	RR(TDF): 0.87(0.77-0.99); RR(IDF): 0.65(0.45-0.88); RR(SDF): 0.78(0.64-0.95); RR(FDF): 0.98(0.85-1.12); RR(VDF): 0.91(0.70-1.18); RR(GDF): 1.03(0.95-1.12)	TDF: I^2=56.6%; IDF: I^2=56.0%; SDF: I^2=0.0%; FDF: I^2=31.4%; VDF: I^2=75.0%; GDF: I^2=0.0%	Per 1 g/day increment in total dietary fiber RR: 0.996 (0.989 – 1.002)	P=0.064		Probable
esophageal cancer	Lingli Sun [23]	15	15CC	3,625/ 16,885	TDF	RR: 0.52 (0.43-0.64)	I^2=71.6%	Per 10 g/day increment in total dietary fiber RR: 0.69 (0.61 – 0.79)	P>0.05		not conclusive
breast cancer	Maryam S. Farvid [16]	20	2CC,17C,1CT	69,735/ 2,092,037	TDF, IDF, SDF, FDF, VDF, GDF, LDF	RR(TDF): 0.92(0.88-0.95); RR(IDF): 0.93(0.86-1.00); RR(SDF): 0.90(0.84-0.96); RR(FDF): 0.93(0.89-0.96); RR(VDF):	TDF: I^2=12.6%; IDF: I^2=33.4%; SDF: I^2=12.6%; FDF: I^2=9.0%; VDF:		P>0.05		Probable

						0.95(0.90-1.00); RR(GDF): 0.97(0.93-1.01); RR(LDF): 0.97(0.92-1.03)	I^2=39.6%; GDF: I^2=29.6%; LDF: I^2=0.0%				
colon cancer	Vincenza Gianfredi [18]	21	8CC,13C	>1,000/ 2,627,391	TDF	RR: 0.74(0.67-0.82)	I^2=43.8%		P=0.177		Probable
Rectal Cancer	Vincenza Gianfredi [17]	22	9CC,13C	>1,000/ 2,876,136	TDF	RR: 0.77(0.66-0.89)	I^2=9.1%		P=0.816		Probable
renal cell carcinoma	Tian-bao Huang [19]	7	4CC,3C	6,115/ 941,202	TDF, IDF, SDF, FDF, VDF, GDF, LDF	RR(TDF): 0.84(0.74- 0.96); RR(FDF): 0.92(0.80-1.05); RR(VDF): 0.70(0.49-1.00); RR(GDF): 1.04(0.91-1.18); RR(LDF): 0.80(0.69-0.93)	TDF: I^2=23.8%; FDF: I^2=0.0%; VDF: I^2=76.9%; GDF: I^2=0.0%; LDF: NA	Per 10 g/day increment in total dietary fiber RR: 0.94 (0.80 – 1.11)	Egger's test and Begg's test (P = 0.728, P = 0.707, respectively)		suggestive
endometrial cancer	Hengjie Li [20]	16	13CC,3C	6,563/ 198,174	TDF	RR: 0.86(0.78-0.93)	TDF: I^2=69.1%		P>0.05		Probable
Colorectal Adenoma	Daniele Nucci [22]	21	12CC,8C,1CT	>1,000/ 157,725	TDF	RR: 0.74(0.67-0.82)	TDF: I^2=62.7%		P=0.838		Probable
Pancreatic Cancer	Daniele Nucci [21]	18	15CC,3C	>1,000/ 343,120	TDF	RR: 0.63(0.53-0.76)	TDF: I^2=68.2%		P=0.006		Probable

ovarian cancer	Hui Xu [24]	19	14CC,5C	8,200/ 567,742	TDF	RR: 0.70(0.57-0.87)	TDF: I^2=83.5%		*P*=0.276		suggestive
Gastric Cancer	Zhizhong Zhang [25]	21	19CC,2C	6,950/ 580,064	TDF	RR: 0.58(0.49-0.67)	TDF: I^2=62.2%	Per 10 g/day increment in total dietary fiber RR: 0.56 (0.45 –0.71)	*P*=0.931		Convincing
